# Structural Investigation of Chloride Ion-Containing Acrylate-Based Imidazolium Poly(Ionic Liquid) Homopolymers and Crosslinked Networks: Effect of Alkyl Spacer and N-Alkyl Substituents

**DOI:** 10.3390/nano15010040

**Published:** 2024-12-29

**Authors:** Mahmoud Al-Hussein, Lisa Ehrlich, Doris Pospiech, Petra Uhlmann

**Affiliations:** 1 Physics Department, The University of Jordan, Amman 11942, Jordan; 2 Leibniz-Institut für Polymerforschung Dresden e.V., Hohe Str. 6, 01069 Dresden, Germany; 3 School of Science, Technische Universität Dresden, 01062 Dresden, Germany

**Keywords:** polymerized ionic liquids, crosslinking, conductive salt, chain packing, internal structure, wide-angle X-ray scattering, ionic conductivity, normalized heterogeneity length

## Abstract

Understanding the interplay between the molecular structure of the ionic liquid (IL) subunit, the resulting nanostructure and ion transport in polymerized ionic liquids (PILs) is necessary for the realization of high-performance solid-state electrolytes required in various advanced applications. Herein, we present a detailed structural characterization of a recently synthesized series of acrylate-based PIL homopolymers and networks with imidazolium cations and chloride anions with varying alkyl spacer and terminal group lengths designed for organic solid-state batteries based on X-ray scattering. The impact of the concentrations of both the crosslinker and added tetrabutylammonium chloride (TBACl) conducting salt on the structural characteristics is also investigated. The results reveal that the length of both the spacer and terminal group influence the chain packing and, in turn, the nanophase segregation of the polar domains. Long spacers and terminal groups seem to induce denser polar aggregates sandwiched between more compact alkyl spacer and terminal group domains. However, the large inter-backbone spacing achieved seems to limit the ionic conductivity of these PILs. More importantly, our findings show that the previously reported general relationships between the ionic conductivity and the structural parameters of the nanostructure of PILs are not always attainable for different molecular structures of the IL side group.

## 1. Introduction

Polymerized ionic liquids (PILs) are a class of materials that combines the characteristics of ILs and polymers including ionic conductivity, high thermal and electrochemical stability and good mechanical properties [1,2,3,4]. They are currently being investigated for advanced materials applications such as solid-state batteries, supercapacitors, fuel cells and electrochromic devices [5,6,7,8,9]. Taking advantage of their flexible molecular design, their physicochemical properties can be readily tuned [10]. In this context, major efforts have focused on enhancing their characteristic slow ion transport, caused by ion hopping instead of free diffusion as in liquids, using various types of ions and engineering the structures of the polymer backbone and side chains [11,12,13,14,15]. However, any molecular modification might influence the nanostructure of the PIL, stemming mainly from the nanophase separation between the nonpolar backbones and the polar substituents. This can have drastic consequences on the PIL’s ability to form channels in which ions can be transported and in turn its potential use as a solid electrolyte. Therefore, in order to achieve an acceptable ionic conductivity, it is essential to understand the interplay between the IL structure, the chain packing and resulting nanostructure, and ion transport in the PIL membranes at a molecular level. Ideally, a rational design of the chemical structure and morphology of the PIL would allow for enhanced ion transport, while maintaining an acceptable mechanical strength to advance its applications.

With this in mind, we synthesized a series of acrylate-based PIL homopolymers with imidazolium cations and chloride anions with varying alkyl spacer and terminal group lengths. Copolymers of the IL monomers with bifunctional crosslinker monomers were also synthesized to evaluate the effect of the concentrations of both the crosslinker and added conducting salt on the physical properties of these PIL networks. The synthesis results, characterization and device properties of these PILs have been reported in our previous studies [16,17]. Notably, the satisfactory performance of battery cells of promising candidates of crosslinked PILs as polymer electrolytes has not yet been reported. Therefore, analyzing the nanostructure and morphology of these PILs in comparison with those of other imidazolium-based PILs reported in the literature is necessary to understand their physical properties.

It has already been reported that the length of the alkyl spacer in linear homopolymers with a methyl substituent and chloride counterions has a significant effect on the self-organization behavior of the PIL [18]. Several PILs with other halogenide counterions, including chloride, bromide and iodide, have also been investigated [19,20,21,22,23]. However, elucidating the effect of varying the lengths of both the spacer and terminal group simultaneously on the self-organizing behavior of the PIL has not been discussed in detail before. The present study aims to investigate in detail the impact of the alkyl length of each spacer and terminal group on the self-assembly of these PILs as a systematic series. To this end, wide-angle X-ray scattering (WAXS) measurements were employed to elucidate the nanostructure and molecular packing of the synthesized homopolymers and network copolymers.

## 2. Samples and Methods

### 2.1. Samples

**Figure 1:**
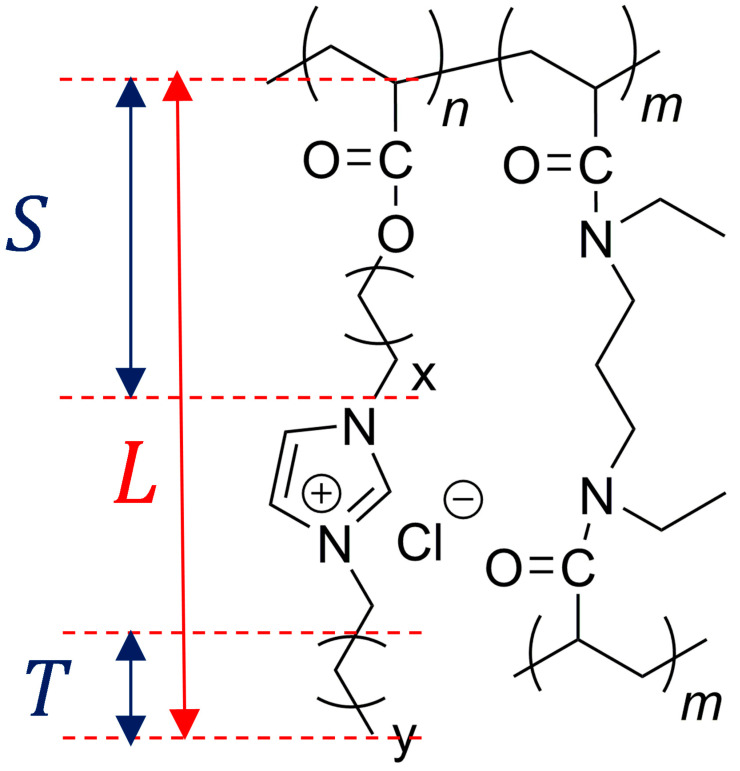
Chemical structure of the synthesized acrylate-based PILs with imidazolium cations and chloride anions (polymers containing only the IL monomer (*n* repeating units): linear homopolymers; copolymers containing the IL monomer with *n* repeating units and the crosslinker monomer with *m* repeating units: networks).

The synthesis of the acrylate-based PILs with imidazolium cations and chloride anions using a two-step procedure followed by purification by dialysis in ethanol was reported in in the work of Ehrlich et al. [16,17]. The chemical structure of the synthesized PILs, abbreviated as p(AAC*_x_*ImC*_y_*Cl), is shown in Figure 1. Variations were made to the alkyl spacer length (C*_x_*), alkyl terminal group length (C*_y_*) for the homopolymers, and crosslinking content for the crosslinked copolymer networks to generate a high degree of flexibility in the structure of the PILs. While *x* varied between propyl (C_3_), hexyl (C_6_) and octyl (C_8_), *y* was either butyl (C_4_) or hexyl (C_6_). Crosslinked copolymers with and without tetrabutylammonium chloride (TBACl) conductive salt are denoted by p(AAC*_x_*ImC*_y_*Cl)/h/k, where h and k indicate the percentage concentration of the crosslinker and conductive salt, respectively. All samples investigated in this study are summarized in Table 1.

The thermal stability, glass transition temperature $T_{g}$, ionic conductivity and electrochemical stability of the samples were characterized by thermogravimetric analysis (TGA), differential scanning calorimetry (DSC), electrochemical impedance spectroscopy (EIS) and linear sweep voltammetry (LSV), respectively [16,17]. A summary of the results obtained in these studies is presented in Table 1.

### 2.2. X-Ray Scattering

To investigate the internal structure and characteristic length scales originating from interactions of ionic, polar and nonpolar moieties of the synthesized homopolymers and crosslinked copolymer PILs, X-ray scattering was used [24,25]. Two-dimensional X-ray scattering patterns were collected using an SAXSLab Ganesha at sample-to-detector distances of 102, 145 and 1502 mm. The instrument was equipped with a Pilatus 300K detector, a Cu X-ray source operated at 50 kV/0.6 mA (λ = 1.5408 Å) and a three-slit collimation system. The scattering patterns were recorded in transmission for 2 h at room temperature under vacuum conditions. The isotropic 2D patterns were radially integrated and then displayed as one-dimensional plots of the intensity as a function of the modulus of the scattering vector, $q=\frac{4\pi }{\lambda }\sin\theta$, where 2θ is the scattering angle. The background was subtracted after normalizing the total scattering profile by the transmitted intensity. Small-angle regions were featureless, therefore, we present here the wide-angle X-ray (WAXS) patterns.

## 3. Results and Discussion

### 3.1. Linear Homopolymers

**Figure 2:**
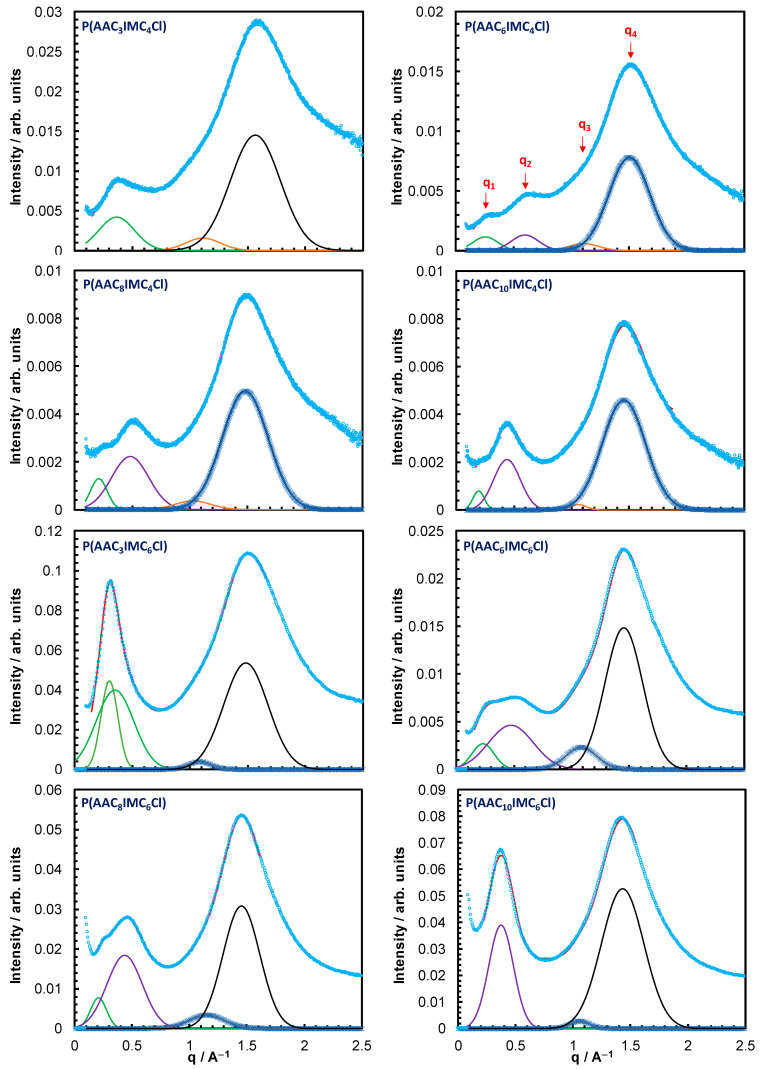
Integrated curves of the 2D WAXS patterns for both p(AAC*_x_*ImC_4_Cl) and p(AAC*_x_*ImC_6_Cl) homopolymers with spacer lengths *x* = 3, 6, 8 and 10. The Gaussian function fits of the broad peaks are also shown.

Figure 2 shows the integrated curves of the 2D WAXS patterns, shown in the Supporting Information (Supplementary Figure S1), for both the p(AAC*_x_*ImC_4_Cl) and p(AAC*_x_*ImC_6_Cl) homopolymers with x = 3, 6, 8 and 10. Careful fitting of the curves revealed that three or four broad peaks can be observed depending on the value of x in the homopolymer. We denote them by $q_{1}$, $q_{2}$, $q_{3}$ and $q_{4}$ with increasing q, as illustrated in the curve of the p(AAC_6_ImC_4_Cl) sample. The appearance of these peaks indicates that all the samples underwent nanophase separation between the anions and polar and nonpolar substituents of the homopolymers. The position and full width at half-maximum (FWHM) for all the peaks were determined by fitting each curve to either three or four Gaussian functions plus a constant background. The repeating distance corresponding to each of these peaks is calculated using Bragg’s law $d_{n}=\frac{2\pi }{q_{n}}$. Multiple experimental and simulation studies have demonstrated that three characteristic peaks are commonly observed in the WAXS patterns of imidazolium-based PILs, corresponding to different intra- and intermolecular correlations [26,27,28,29,30,31]. The low-*q* peak corresponds to the polymer backbone-to-backbone repeating distance, whereas the intermediate-*q* and high-*q* peaks are assigned to the anion–anion and side chain–side chain correlations, respectively.

As observed in Figure 2 and Table 1, for all the homopolymers, the $q_{1}$ peak showed a notable dependence on *x*, with the exception of p(AAC_10_ImC_6_Cl). Its position moved gradually to lower *q* values with increasing *x* for both homopolymers. Such behavior is characteristic for the low-*q* peak, as communicated in other reports for imidazolium-based PILs. Therefore, $q_{1}$ is assigned to the polymer backbone-to-backbone repeating distance, $d_{b}$, in accordance with the literature [26,27,28,29,30,31]. In contrast, the positions of both the $q_{3}$ and $q_{4}$ peaks remained essentially constant for all the homopolymers. Therefore, the weak peak $q_{3}$ and the more pronounced peak $q_{4}$ are assigned to the repeating distances of anion–anion, $d_{a}$, and side chain–side chain, $d_{s}$, respectively, in line with previous experimental and simulation studies. It is also interesting to note that the intensity of $q_{1}$ has substantially diminished for the p(AAC_10_ImC_4_Cl) sample and completely disappeared for p(AAC_10_ImC_6_Cl). This might be due to an increasingly reduced contrast in electron density between the polar domains and segregated backbones of the longer side chains, as will be explained below.

**Figure 3:**
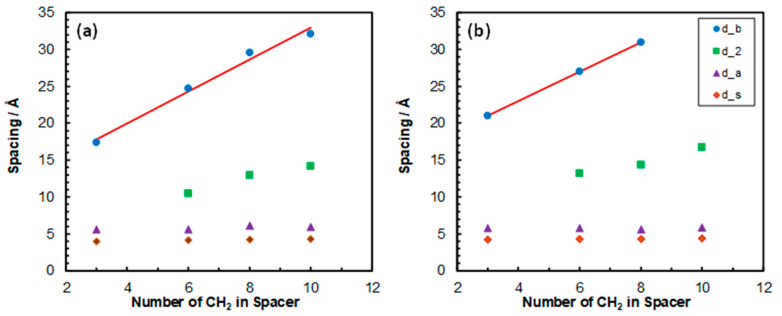
Variations in backbone-to-backbone ($d_{b}$), anion–anion ($d_{a}$) and side chain-to-side chain ($d_{s}$) distances with number of CH_2_ groups in the spacer for (**a**) p(AAC*_x_*ImC_4_Cl) and (**b**) p(AAC*_x_*ImC_6_Cl) homopolymers. The red solid line is a linear fit.

For further analysis of the nanostructure, the variations in the three repeating distances with the change in *x* are plotted in Figure 3 for both homopolymers. As can be seen, $d_{b}$ increased linearly, while both $d_{a}$ and $d_{s}$ did not vary much with *x*. The rate of increase in $d_{b}$ per CH_2_ group is 2.1 and 2.0 Å for p(AAC*_x_*ImC_4_Cl) and p(AAC*_x_*ImC_6_Cl), respectively. These values are close to what have been reported in other studies for imidazolium-based PILs [27,31,32].

**Figure 4:**
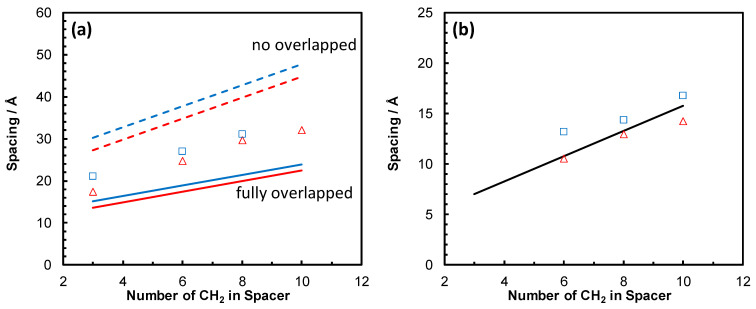
(**a**) Variations in $d_{b}$ with number of CH_2_ groups in the spacer for p(AAC*_x_*ImC_4_Cl) (open triangles) and p(AAC*_x_*ImC_6_Cl) (open squares) homopolymers. The solid lines are the calculated values of the corresponding extended length of the whole side chain (*L*) for p(AAC*_x_*ImC_4_Cl) (red) and p(AAC*_x_*ImC_6_Cl) (blue). The dashed lines are corresponding values of twice the extended length of the whole side chain (2*L*) for p(AAC*_x_*ImC_4_Cl) (red) and p(AAC*_x_*ImC_6_Cl) (blue). (**b**) Variations in $d_{2}$ with number of CH_2_ groups in the spacer for p(AAC*_x_*ImC_4_Cl) (open triangles) and p(AAC*_x_*ImC_6_Cl) (open squares). The solid line represents the corresponding values of the extended length of the spacer (*S*).

Moreover, by comparing the measured values of $d_{b}$ with their corresponding extended length of the whole side chain including the imidazolium ring (*L*), an overlap (partial or complete interdigitation) of the side chains can be evaluated. As shown in Figure 4, all the samples exhibited only partial interdigitation that decreased with increasing *x*. Long spacers and terminal groups chains have higher steric demand and thus restrict the overlap of opposing side chains. It is worth noting that these trends are opposite to those presented in previous reports on imidazolium-based PILs with alkyl tails [11,18,27,31]. However, those studies have investigated the influence of the side chain length on the nanostructure by merely varying the alkyl tail length without additional spacer. Therefore, the Columbic attractions decrease while the van der Waals interactions increase, leading to less segregated polar domains for longer side chains in these systems. In contrast, the side chain length is varied by changing both the spacer and terminal group lengths in our samples. This decoupling appears to lessen the competition between the polar groups’ electrostatic interactions and the side chains’ van der Waals interactions and lead to more segregation of the polar substituents for longer spacer and terminal groups. This entails an increasing electron density of the more segregated polar domains to comparable values of the segregated backbones for the longer side chains and in turn a lower contrast in their corresponding electron densities. This is evidenced by the increasingly diminishing intensity of $q_{1}$ for the p(AAC_10_ImC_4_Cl) and p(AAC_10_ImC_6_Cl) samples.

**Figure 5:**
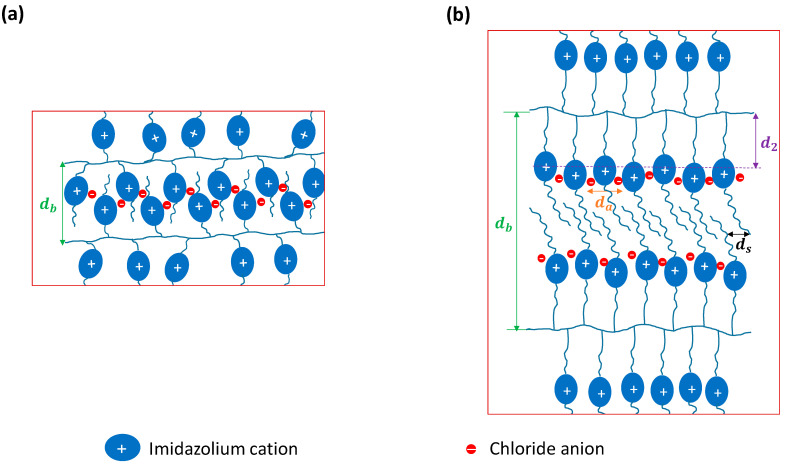
Schematic representation of the structure of the PIL homopolymers with (**a**) short spacers and terminal groups and (**b**) long spacers and terminal groups.

A question that arises now is as follows: what is the origin of the newly appearing fourth peak $q_{2}$ characteristic of our samples, which has not been discussed before? In an attempt to answer this relevant question, the variations in its spacing, $d_{2}$, as well as the distance starting from the backbone to the imidazolium ring, *S*, with *x* were plotted in Figure 4b. Interestingly, all the samples exhibited a close match between $d_{2}$ and the corresponding *S* values for both homopolymers. This seems to suggest that $q_{2}$ is related to the correlation in the electron density between the backbones and the associated imidazolium-chloride ionic aggregates within the backbone–backbone alternations, as illustrated schematically in Figure 5. Furthermore, it can be concluded from these results that the appearance of the fourth peak is directly related to spacers with sufficient lengths as well as long terminal groups. The imidazolium cations and Cl anions associate due to the electrostatic interaction while the alkyl spacer and terminal groups are driven to pack together and are stabilized by van der Waals interactions, resulting in an alternating polar–nonpolar structure. This gives rise to an additional modulation in the electron density due to the alkyl spacer–polar aggregate–alkyl terminal groups within the backbone-to-backbone repeating distance; see Figure 5. Longer spacer and terminal groups give rise to more effective van der Waals interactions and, in turn, more compact spacer and terminal group domains sandwiching the polar aggregates. This results in the better segregation of the polar domains, enhancing their density, which eventually becomes close to the backbone domain density. This can also explain the increasingly reduced intensity of $q_{1}$ with *x*. The fact that $q_{2}$ did not show up as a distinct peak in the results for p(AAC_3_ImC_4_Cl) and p(AAC_3_ImC_6_Cl) homopolymers, which have the shortest spacers, points to less compact polar domains due to the short spacer.

In summary, these results clearly demonstrate that the length of both the spacer and terminal groups influence the chain packing and, in turn, the segregation of the polar domains of the synthesized imidazolium-based PILs. Long spacer and terminal groups seem to induce denser polar aggregates in addition to larger inter-backbone spacing.

### 3.2. PIL Networks

**Figure 6:**
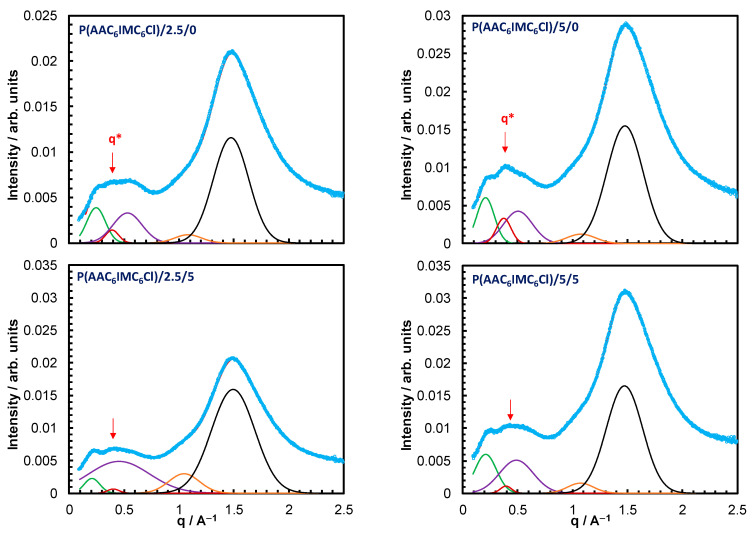
The integrated curves of the 2D WAXS patterns for p(AAC_6_ImC_6_Cl)/2.5/0, p(AAC_6_ImC_6_Cl)/5/0, p(AAC_6_ImC_6_Cl)/2.5/5 and p(AAC_6_ImC_6_Cl)/5/5 copolymer networks. The Gaussian function fits of the broad peaks are also shown.

We focus here on the crosslinked p(AAC_6_ImC_6_Cl) copolymer sample, as it exhibited the most mechanically stable electrolytes for the battery applications. Figure S2 and Figure 6 show the WAXS patterns and the corresponding integrated curves for the p(AAC_6_ImC_6_Cl)/2.5/0 and p(AAC_6_ImC_6_Cl)/5/0 copolymer networks with 2.5 and 5 mol% crosslinker concentrations, respectively.

Interestingly, a new fifth peak, $q^{*}$, with a d-spacing of around 16 Å appears in the curves of both copolymer networks. This shows that crosslinking influences the side chain packing and in turn modulates the electron density, giving rise to an additional correlation between the backbones and the polar aggregates at this spacing. Meanwhile, the $q_{2}$ peak characteristic of the p(AAC_6_ImC_6_Cl) homopolymer is still observed in these crosslinked copolymers, indicating that some of the original non-crosslinked alternating structure still exists in the crosslinked copolymers. This suggests a heterogeneous structure of these crosslinked copolymers with two types of the alternating structures.

Shown also in Figure S3 and Figure 6 are the WAXS patterns and corresponding integrated curves for the two crosslinked copolymers with 5 mol% concentration of TBACl conducting salt. The notable increase in the backbone-to-backbone distance of the p(AAC_6_ImC_6_Cl)/2.5/5 sample (with 5 mol% conducting salt) implies the incorporation of the salt molecules within the polar aggregates of the alternating structure. The incorporated salt molecules are expected to increase the steric hindrance, leading to less compact interdigitation between the side chains of adjacent backbones. However, upon adding 5 mol% salt to the p(AAC_6_ImC_6_Cl)/5/5 sample, $d_{b}$ did not increase further, indicating that the excessive salt molecules cannot be accommodated by the alternating structure.

### 3.3. Correlation Between Conductivity and Internal Structure

The ionic conductivity in the PILs is determined by both the density and mobility of the charge carriers [30,33]. The ion mobility in its turn is directly related to charge carrier diffusion, which is mainly realized in polymer materials by ion hopping between polar domains. Both elastic forces and electrostatic interactions can affect ion transport in PILs. Consequently, the nanoscale morphology of PILs can play a key role in determining the ionic conductivity. There have been several studies conducted in attempt to identify a general relationship between the conductivity and nanostructure of PILs. Among these studies, Salas-dela Cruz et al. suggested that conductivity can be directly related to the backbone-to-backbone distance [31]. In another work by Doughty et al., the structural heterogeneities of the nanophase separated domains were found to be more influential [27]. According to their findings, heterogeneous structures have lower activation barriers for ions to jump between polar domains and thus the PIL has enhanced ionic conductivity.

We will now try to analyze our results in terms of these ideas. The values of the measured room temperature conductivity and $T_{g}$ for all the samples are listed in Table 1. As can be observed from this table, essentially, the ionic conductivity values of the p(AAC_x_ImC_4_Cl) homopolymers decrease with increasing $d_{b}$, in agreement with Salas-dela Cruz et al. and other observations. P(AAC_8_ImC_4_Cl) is an exception though. Note that this sample has the lowest $T_{g}$ (−51 °C) among all of the homopolymers. The exact reason that could cause such behavior is not clear for us at the moment. It might be due to the presence of residual monomers or residual solvents (although all the polymers were purified by dialysis) that counterbalance the increased $d_{b}$ effect. In contrast, the p(AAC_x_ImC_6_Cl) homopolymers with longer terminal groups did not show any clear relation between ionic conductivity and $d_{b}$. Therefore, we can conclude that a general relationship between conductivity and $d_{b}$ cannot be defined for the samples under investigation.

We now turn to evaluating the potential correlation between structural heterogeneities and ionic conductivity for our samples. The normalized heterogeneity length corresponding for fluctuations in $d_{b}$ can be calculated by analyzing both the position and width of the $q_{1}$ peak. First, the correlation length, $\Gamma_{b}$, is calculated by $\Gamma_{b}=\frac{2\pi }{\Delta q_{1}}$, where $\Delta q_{1}$ is the FWHM of the $q_{1}$ peak [27]. Then, the normalized heterogeneity length, Λ, is determined by $\Lambda =\frac{\Gamma_{b}}{d_{b}}=\frac{q_{1}}{\Delta q_{1}}$. Table 2 summarizes the calculated values. A large normalized heterogeneity length reflects an ordered structure, whereas a small value indicates fluctuations in the $d_{b}$ distances and a less ordered structure. As can be observed from Table 2, an increase in the length of the spacer (*x*) correlates with an increase in the heterogeneity length for the p(AAC*_x_*ImC_4_Cl) homopolymers, in line with the observations of Doughty et al. [27]. However, the p(AAC*_x_*ImC_6_Cl) homopolymers exhibited the opposite trend. It is interesting to note from Table 1 and Table 2 that for the p(AAC*_x_*ImC_4_Cl) homopolymers, the p(AAC_3_ImC_4_Cl) sample, which has the shortest spacer, exhibits the highest conductivity and the most disordered structure (lowest normalized heterogeneity length of ~1.00). In a striking difference, for the p(AAC*_x_*ImC_6_Cl) homopolymers, the ionic conductivity is the lowest for the sample with the shortest spacer, namely, p(AAC_3_ImC_6_Cl), which has the largest normalized heterogeneity length (1.74). Meanwhile, both the p(AAC_6_ImC_6_Cl) and p(AAC_8_ImC_6_Cl) samples have a comparable normalized heterogeneity length (~1.00). Nonetheless, p(AAC_6_ImC_6_Cl) has a smaller $d_{b}$ and much higher conductivity. This can also be noted for the p(AAC_3_ImC_4_Cl) and p(AAC_6_ImC_4_Cl) samples. Both of the homopolymers have a similar normalized heterogeneity length (~1.00), but p(AAC_3_ImC_4_Cl) has a smaller $d_{b}$ and higher conductivity. Therefore, it seems that for the samples with comparable normalized heterogeneity lengths, shorter inter-backbone distances promote higher conductivity. It is also worth noting that the p(AAC_10_ImC_6_Cl) homopolymer exhibited a remarkable conductivity ($3.18\times 10^{-4}$ S/cm). Note also that $q_{1}$ disappeared for this sample and, therefore, the normalized heterogeneity could not be calculated. These cumulative results indicate that, in contrast to Doughty et al.’s findings, there is no simple general relationship between the normalized heterogeneity and conductivity for our samples.

Notably, both the crosslinked copolymers (p(AAC_6_ImC_6_Cl)/2.5/0 and p(AAC_6_ImC_6_Cl)/5/0) exhibited lower conductivities than their homopolymer counterparts. The normalized heterogeneity length increased from 1.01 for the homopolymer to 1.22 and 1.12 for 2.5 and 5 mol% crosslinked copolymers, respectively. More interestingly, $d_{b}$ first decreased from 27.00 Å for the homopolymer to 25.73 Å for the 2.5 mol% crosslinked sample, but then it increased to 30.03 Å for the 5 mol% crosslinked sample. Adding 5% conducting salt to both crosslinked copolymers resulted in a substantial decrease in the conductivity for the p(AAC_6_ImC_6_Cl)/2.5/5 copolymer, accompanied by a pronounced increase in $d_{b}$ from 25.73 to 29.99 Å. As for p(AAC_6_ImC_6_Cl)/5/5, there was a slight increase in conductivity after adding the conductive salt with a decrease in the normalized heterogeneity length from 1.12 to 0.92 and almost the same $d_{b}$ of the p(AAC_6_ImC_6_Cl)/5/0, as discussed before.

In conclusion, these findings clearly show that simple correlations between the ion transport and the structural parameters of the nanostructure of PILs are not always attainable for different molecular structures of the IL side group. Such complex behavior stems from the fact that conductivity in PILs is a complex phenomenon influenced by the multi-length and time scales of the chain organization and associated polar constituents’ local environment as well as the preparation conditions of the samples used for ionic conductivity measurements.

## 4. Conclusions

We have studied the molecular packing and nanostructure of a series of acrylate-based PILs (both as linear homopolymers and as copolymer networks) with imidazolium cations and chloride anions with varying alkyl spacer and terminal group lengths using X-ray scattering. The alkyl spacer length varied between propyl (C_3_), hexyl (C_6_) and octyl (C_8_), while the terminal group was either butyl (C_4_) or hexyl (C_6_). The effect of the concentration of both the crosslinker and tetrabutylammonium chloride added as an ion conducting salt on the nanostructure was also evaluated, taking into account that nanostructuration may enhance the ionic conductivity of the materials.

The results revealed that the length of both the alkyl spacer and alkyl terminal group influenced the chain packing and, in turn, the segregation of the polar constituents. Long spacers and terminal groups induced enhanced segregation of the polar substituents. We attribute this behavior to decoupling the self-assembly of the alkyl spacer and terminal group into separate domains, squeezing the polar aggregates. This decoupling seems to reduce the competition between the cation–anion electrostatic interactions and the alkyl group van der Waals interactions in contrast to other previously reported imidazolium-based PILs.

We also tried to correlate the determined structure characteristics with the ionic conductivity of both the homopolymers and crosslinked copolymers following recently reported relationships for other imidazolium-based PILs. However, no obvious relationships between the different structural length scales of the nanostructure (the backbone-to-backbone distance and the normalized heterogeneity length) and ionic conductivity were found for our samples. Our findings demonstrate that previously reported simple correlations between ion transport and the structural parameters of the nanostructure are not always attainable for homopolymers and crosslinked networks of PILs, having only slight variations in the chemical structure of their IL side groups. They also contribute to the understanding and control the relationship between the molecular structure of the IL group, the resulting nanostructure and ion transport in PILs, necessary for the realization of high-performance solid-state batteries.

## Figures and Tables

**Table 1:** Summary of the characteristics of the different PIL homopolymers and crosslinked copolymer networks, as reported in refs. [16,17].

Sample	$T_{g}$ (1st Heating) (°C)	Ionic Conductivity at 22.4 °C (S/cm)
p(AAC_3_ImC_4_Cl)	−22	$2.33\times 10^{-4}$
p(AAC_6_ImC_4_Cl)	−39	$1.31\times 10^{-5}$
p(AAC_8_ImC_4_Cl)	−51	$1.38\times 10^{-4}$
p(AAC_10_ImC_4_Cl)	−35	$3.16\times 10^{-6}$
p(AAC_3_ImC_6_Cl)	−29	$4.44\times 10^{-9}$
p(AAC_6_ImC_6_Cl)	−23	$2.15\times 10^{-6}$
p(AAC_8_ImC_6_Cl)	−36	$8.89\times 10^{-8}$
p(AAC_10_ImC_6_Cl)	−6	$3.18\times 10^{-4}$
p(AAC_6_ImC_6_Cl)/2.5/0	−28	$9.67\times 10^{-7}$
p(AAC_6_ImC_6_Cl)/5/0	−27	$4.52\times 10^{-7}$
p(AAC_6_ImC_6_Cl)/2.5/5	−30	$2.03\times 10^{-7}$
p(AAC_6_ImC_6_Cl)/5/5	−30	$6.03\times 10^{-7}$

**Table 2:** Calculated d-spacings of $q_{1}$ and $q_{2}$ peaks ($d_{b}$ and $d_{2}$, respectively), FWHM of $q_{1}$ ($\Delta q_{1})$ and normalized heterogeneity length (Λ) for the indicated samples.

Sample	$d_{b}$ (Å)	$\Delta q_{1}$ (Å^−1^)	$d_{2}$ (Å)	Λ
p(AAC_3_ImC_4_Cl)	17.37	0.363	–	0.996
p(AAC_6_ImC_4_Cl)	24.70	0.249	10.51	1.02
p(AAC_8_ImC_4_Cl)	29.62	0.170	12.96	1.25
p(AAC_10_ImC_4_Cl)	32.14	0.118	14.23	1.66
p(AAC_3_ImC_6_Cl)	21.05	0.172	–	1.74
p(AAC_6_ImC_6_Cl)	27.00	0.231	13.20	1.01
p(AAC_8_ImC_6_Cl)	31.02	0.196	14.38	1.03
p(AAC_10_ImC_6_Cl)	–	–	16.76	–
p(AAC_6_ImC_6_Cl)/2.5/0	25.73	0.199	11.85	1.22
p(AAC_6_ImC_6_Cl)/5/0	30.03	0.186	12.45	1.12
p(AAC_6_ImC_6_Cl)/2.5/5	29.99	0.262	13.86	0.798
p(AAC_6_ImC_6_Cl)/5/5	29.75	0.229	12.82	0.920

## Data Availability

The data are contained within the article and Supplementary Materials.

## Supplementary Materials

The following supporting information can be downloaded at https://www.mdpi.com/article/10.3390/nano15010040/s1. Figure S1: Two-dimensional WAXS patterns of the p(AAC*_x_*ImC_4_Cl) homopolymers; Figure S2: Two-dimensional WAXS patterns of the p(AAC*_x_*ImC_6_Cl) homopolymers; Figure S3: Two-dimensional WAXS patterns of crosslinked copolymers with and without added TBACl conductive salt.
