# Supplementary material for: Structural Investigation of Chloride Ion-Containing Acrylate-Based Imidazolium Poly(Ionic Liquid) Homopolymers and Crosslinked Networks: Effect of Alkyl Spacer and N-Alkyl Substituents
Source: Nanomaterials (Basel). 2024 Dec 29;15(1):40. doi: 10.3390/nano15010040 (PMC11722141; doi:10.3390/nano15010040)
Supplement: Supplementary file 1 [file nanomaterials-15-00040-s001.zip › nanomaterials-3350311-supplementary.pdf]

Supplementary Information

Structural Investigation of Chloride Ion-Containing Acrylate-Based Imidazolium Poly(Ionic Liquid) Homopolymers and Crosslinked Networks: Effect of Alkyl Spacer and N-Alkyl Substituents

Mahmoud Al-Hussein [1,*], Lisa Ehrlich [2,3], Doris Pospiech [2] and Petra Uhlmann [2,*]

[1] The University of Jordan, Physics Department, Amman 11942, Jordan
[2] Leibniz-Institut für Polymerforschung Dresden e.V., Hohe Str. 6, 01069 Dresden, Germany
[3] Technische Universität Dresden, School of Science, Dresden, Germany
* Correspondence: m.alhussein@ju.edu.jo (M.A.H.); uhlmannp@ipfdd.de (P.U.)

2D WAXS Patterns

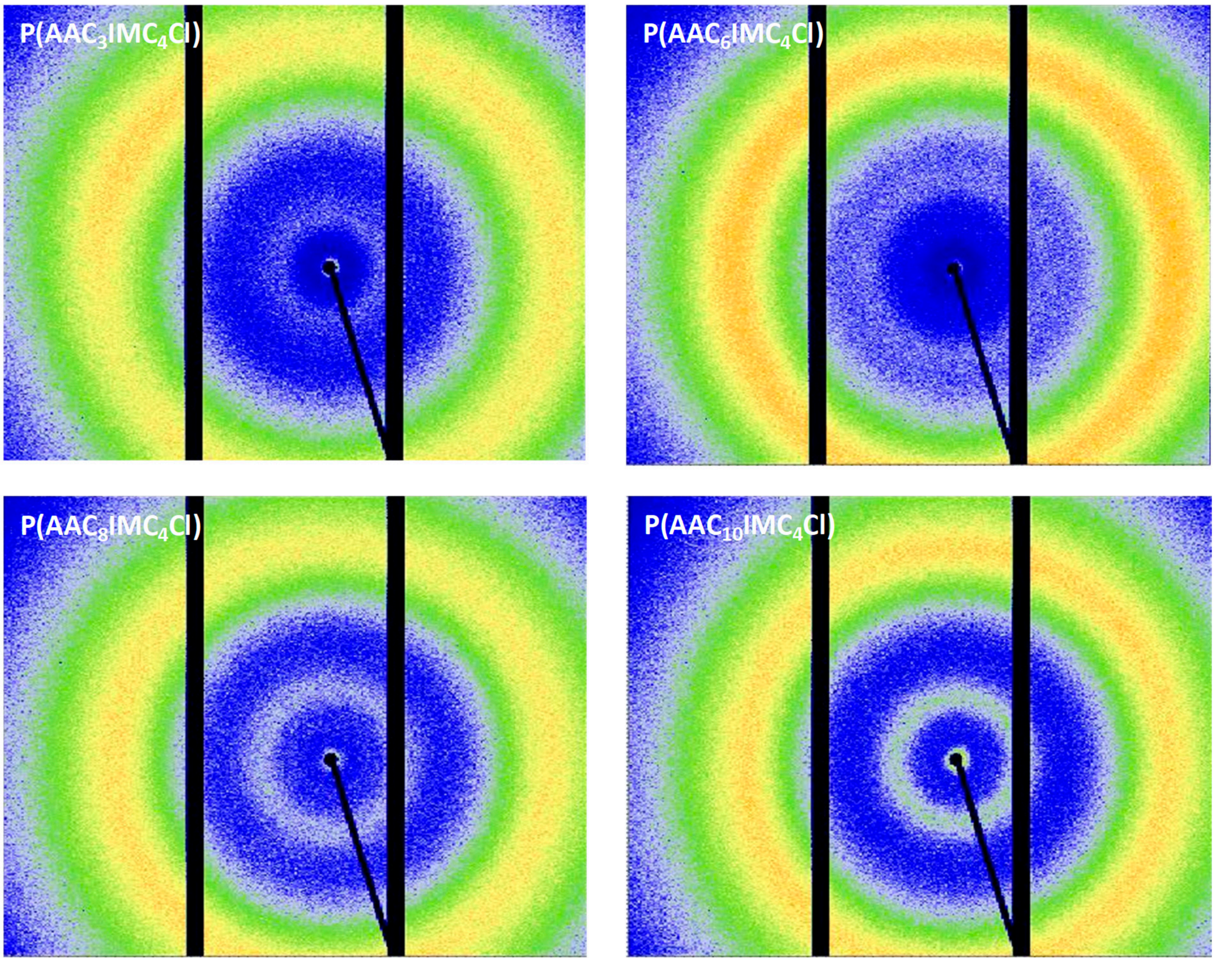

Figure S1: 2D WAXS patterns of the p(AAC$_x$ImC$_4$Cl) homopolymers.

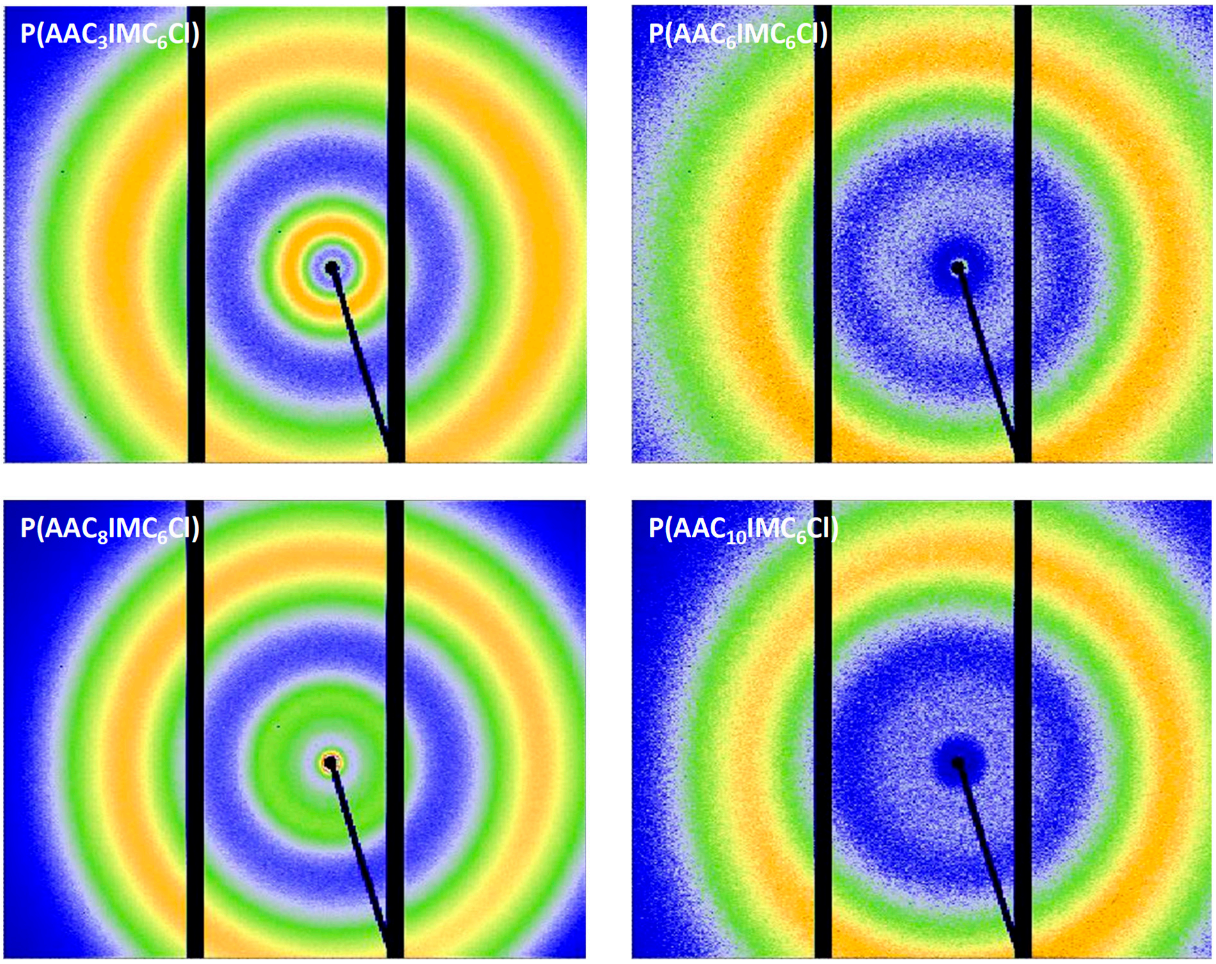

Figure S2: 2D WAXS patterns of the p($AAC_x ImC_6 Cl$) homopolymers.

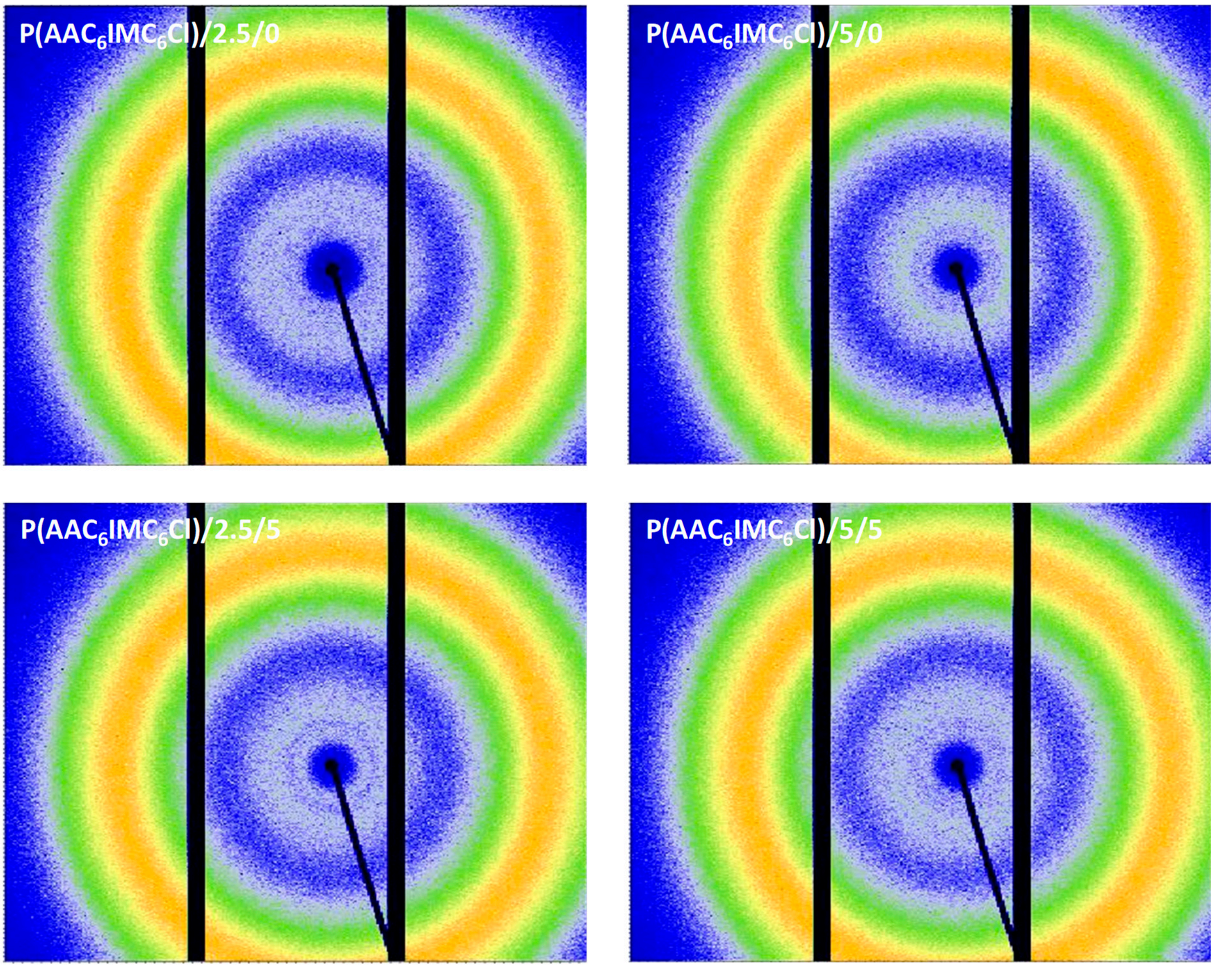

Figure S3: 2D WAXS patterns of crosslinked copolymers with and without added TBACl conducting salt.
